# Defensive medicine practices and workplace violence among physicians in tertiary healthcare settings in Zagazig, Egypt: a cross-sectional study

**DOI:** 10.1186/s12889-026-29209-9

**Published:** 2026-09-08

**Authors:** Aya M. Abdelhamid, Amal E Gohary, Aya M. Fathi, Reham Zakaria, Sara Mohamed Salem, Al Shymaa Mohamed Abd El Hadi, Mohamed Gomaa Abdeen, Amany I. A. Soliman

**Affiliations:** 1 Department of Family Medicine, Faculty of Medicine, Zagazig University, Zagazig, 44519 Egypt; 2 Department of Pediatrics, Faculty of Medicine, Zagazig University, Zagazig, 44519 Egypt; 3 Department of Public Health and Environmental Medicine, Faculty of Medicine, Zagazig University, Zagazig, 44519 Egypt; 4 Department of General Surgery, Faculty of Medicine, Zagazig University, Zagazig, 44519 Egypt; 5 Department of Internal Medicine, Faculty of Medicine, Zagazig University, Zagazig, 44519 Egypt; 6 Department of Obstetrics and Gynaecology, Faculty of Medicine, Zagazig University, Zagazig, 44519 Egypt; 7 Department of Urology, Faculty of Medicine, Zagazig University, Zagazig, 44519 Egypt

**Keywords:** Defensive medicine, Workplace violence, Medical litigation, Physicians, Egypt, Surgical specialties

## Abstract

**Background:**

Defensive medicine involves clinical practices—either through overuse of interventions (positive) or avoidance of high-risk procedures (negative)—driven by liability concerns rather than patient care. In Egypt, rising medico-legal issues and workplace violence may exacerbate these behaviors. This study assessed the prevalence and drivers of defensive medicine among physicians at a tertiary academic hospital, comparing medical and surgical specialties and examining the impact of prior litigation experience.

**Methods:**

A cross-sectional study was conducted at Zagazig University Hospitals using a two-stage sampling design, involving stratified random selection of departments followed by voluntary response sampling of physicians through an online questionnaire. Data were collected between 1 November 2024 and 30 April 2025 through a self-administered questionnaire covering: (1) socio-demographic and occupational characteristics; (2) positive and negative defensive medicine practices during physicians’ entire career; and (3) perceived causes of defensive practices and involvement in medical litigation. Data were analyzed using Chi-square and Fisher’s exact tests. Binary logistic regression analysis was performed to detect the associations of age, sex, years in profession, working hours/day, and exposure to workplace violence with defensive medicine practice. Statistical significance was set at *p* < 0.05.

**Results:**

The study included 340 physicians, of whom 323 (95%) reported performing at least one defensive medicine practice. Exposure to workplace violence was reported by 261 participants (76.8%) over the past 12 months. The most frequently cited reasons for defensive medicine were fear of medical litigation (234, 68.8%) and fear of disciplinary sanctions (169, 49.7%), whereas prior personal involvement in litigation was less commonly reported (87, 25.6%). Significant specialty-based differences were observed. Surgeons (47.9% of the sample) were more likely than medical specialists to perform unnecessary procedures (*p* = 0.04), avoid high-risk patients (*p* = 0.03), and avoid high-risk procedures (*p* = 0.004). Conversely, medical specialists showed greater willingness to accept litigation-involved patients (*p* = 0.005).

**Conclusion:**

Defensive medicine practices were highly prevalent among physicians in this tertiary academic setting. The findings reflect associations between exposure to workplace violence and defensive behaviors, highlighting the potential influence of medico-legal pressures and unsafe working conditions on clinical decision-making.

**Supplementary Information:**

The online version contains supplementary material available at https://doi.org/10.1186/s12889-026-29209-9.

## Introduction

Defensive medicine refers to clinical actions undertaken primarily to avoid liability rather than to advance patients’ clinical interests [1]. It typically manifests in two forms. Positive defensive medicine involves the overutilization of healthcare resources—such as ordering non-indicated diagnostic tests, prescribing unnecessary treatments, or performing procedures without clear medical justification—to demonstrate thoroughness and reduce the likelihood of legal complaints [1]. In contrast, negative defensive medicine reflects physicians’ avoidance of medically appropriate but high-risk interventions, including refraining from managing complex cases or declining invasive procedures that may benefit patients but carry a higher perceived legal risk [1, 2].

Globally, malpractice claims against healthcare practitioners have risen steadily over the past decade, driven in part by increased patient awareness of legal rights and greater accountability within healthcare systems [3]. In Egypt, the Committee of Medical Ethics of the Egyptian Medical Syndicate receives hundreds of malpractice complaints annually, signalling heightened medico‑legal scrutiny of physicians’ clinical decisions [4].

In the absence of comprehensive institutional legal protection or malpractice coverage, individual physicians may face litigation alone, exposing them to severe professional and legal consequences, such as license suspension or criminal liability [5]. Within Egypt’s predominantly public and resource‑strained healthcare system—characterized by workforce shortages and limited structural support—such pressures may intensify physicians’ perceived vulnerability to legal action. Consequently, defensive medical practices may emerge as a self‑protective strategy to mitigate potential medico‑legal repercussions [3, 6].

Simultaneously, workplace violence (WPV) against healthcare workers has been increasingly documented in Egypt and worldwide [7–9]. Exposure to verbal abuse, threats, or physical aggression has been associated with heightened fear, psychological distress, and risk-averse clinical decision-making, potentially contributing to defensive medical practices [10]. Empirical evidence also suggests that WPV may influence healthcare utilization patterns, including increased diagnostic testing, prolonged hospital stays, and reduced use of invasive procedures, all of which are consistent with defensive clinical behavior [10, 11].

Defensive medicine may therefore represent a response to the combined pressures of medico-legal uncertainty and unsafe workplace conditions. Although intended to reduce perceived professional risk, such behaviors may result in unnecessary resource utilization, fragmented care, and adverse effects on patient outcomes [11].

Previous studies in Egypt have examined defensive medicine among junior physicians and within selected specialties, focusing primarily on fear of litigation or workplace violence as separate determinants of defensive behavior [2, 3, 9, 10]. However, evidence remains limited regarding the combined influence of these factors across a broad range of medical and surgical specialties within a tertiary academic healthcare setting.

Furthermore, most available evidence was generated under the regulatory framework that existed before the introduction of the Egyptian Medical Liability Law (https://sis.gov.eg/en/media-center/news/the-cabinet-approves-draft-law-on-regulating-medical-liability-and-protecting-patients/). Assessing defensive medicine practices during this transitional period may provide useful baseline information for future evaluations of the potential impact of legislative changes on physicians’ clinical behavior. Therefore, this study aimed to assess the prevalence of defensive medicine practices among physicians working in a tertiary academic hospital in Egypt and to examine their association with workplace violence, medico-legal concerns, and specialty-related characteristics across a range of medical and surgical departments.

## Methods

### Study design, setting, and participants

This cross-sectional study was conducted from November 2024 to April 2025 at Zagazig University Hospitals, a major tertiary academic center in Egypt. We recruited a stratified sample of physicians across medical and surgical departments. Eligible participants included staff physicians and residents with at least one year of clinical experience; those on extended leave or with incomplete responses were excluded. The study adhered to the Declaration of Helsinki, with ethical approval granted by the Zagazig University Institutional Review Board (ZU-IRB #1032). All participants provided written informed consent before data collection.

### Sample size and sampling technique

The sample size was calculated using Epi Info statistical software version 6. The calculation was based on the total number of physicians working in the university hospitals (*n* = 3,347) and a previously reported prevalence of 56.4% for physicians who disclosed prescribing unnecessary medications as a defensive medicine practice [10], with a 95% confidence level. Accordingly, the required sample size was 340 physicians.

A two-stage sampling approach was applied. Physicians were first stratified according to department type (medical and surgical). The Faculty of Medicine at Zagazig University contains 12 medical departments and 11 surgical departments; from each stratum, six departments were randomly selected to ensure a representative distribution of various medical and surgical sub-specialties while maintaining feasibility. The selected surgical departments were General Surgery, Urology, Orthopedic Surgery, Ear, Nose, and Throat (ENT), Anesthesia, and Obstetrics and Gynecology. The selected medical departments were Internal Medicine, Cardiology, Dermatology, Family Medicine, Pediatrics, and Psychiatry. Within the randomly selected departments, physicians were invited to participate via an online form published on the departments’ official platforms. Participation was voluntary (voluntary response sampling), with proportional allocation based on the total number of physicians in each department, based on the following formula: $$\mathrm{Calculated}\;\mathrm{ratio}\;\mathrm{for}\;\mathrm{each}\;\mathrm{department}\;$$$$=\;(\mathrm{Number}\;\mathrm{of}\;\mathrm{physicians}\;\mathrm{in}\;\mathrm{the}\;\mathrm{department})$$$$/\;(\mathrm{Total}\;\mathrm{number}\;\mathrm{of}\;\mathrm{physicians}\;\mathrm{in}\;\mathrm{all}\;\mathrm{selected}\;\mathrm{departments})$$. For example, the number of physicians in the General Surgery department was 230, and the number of physicians in the selected 12 departments was 1740. So, the ratio for the required number of general surgeons from the total sample = 230/1740 = 0.13, and the required number to participate in the study was 0.13 × 340 that equals 45 surgeons.

Participation was voluntary; however, proportional allocation was applied by monitoring responses from each department and closing recruitment once the required sample size for each department was achieved.

### Data collection tools

Data were collected using a structured, self-administered questionnaire adapted from previously published instruments by Arafa et al. [10] and Catino [12]. The questionnaire items were adopted from previously used instruments with no substantive modifications. Although some source questionnaires had not undergone formal psychometric validation, content validity was established using a structured expert-based validation approach. A multidisciplinary panel of experts in public health, family medicine, and clinical specialties systematically evaluated each item for relevance, clarity, and contextual appropriateness. The evaluation process focused on ensuring that the adapted items adequately captured the intended constructs within the local healthcare context. Based on their feedback, the item “have you been physically and/or verbally attacked in your workplace” was edited to “have you been exposed to physical and/or verbal violence in your workplace”. Also, modification was done to the response format of the question " What factors influenced the behaviors indicated above” as the original item-by-item dichotomous response options “agree/disagree” were replaced with a multiple-response format, and the instruction “more than one answer may be selected” was added allowing participants to indicate all applicable factors. Feedback from the expert panel was incorporated iteratively, and necessary refinements were made before pilot testing. It consists of three sections.

Section 1 socio-demographic and occupational data were assessed, including age, sex, marital status, specialty, years of experience, and working hours.

 Section 2 Workplace violence and defensive medicine practices were assessed as two distinct constructs.

Workplace violence was defined as any act or threat of physical violence, harassment, intimidation, or other threatening behavior occurring in the workplace, ranging from verbal abuse to physical assault [13]. WPV exposure was assessed over the preceding 12 months to enhance recall accuracy and align with standard epidemiological practices. This was measured using a single self-reported item asking whether participants had experienced physical or verbal violence during clinical practice (yes/no). The item was adapted from the Workplace Violence in the Health Sector – Country Case Study Research Instruments, based on the World Health Organization and International Labour Organization framework [14].

Defensive medicine practices—both positive and negative—were evaluated as behaviors occurring at any point during the participants’ professional careers, aiming to capture cumulative exposure. For this study, defensive medicine was operationally defined as clinical actions undertaken without clear medical necessity or avoidance of medically indicated high-risk procedures due to fear of legal consequences. Positive defensive medicine practices were assessed using four items addressing unnecessary investigations, interventions, referrals, and medications. Negative defensive practices were assessed using two items related to the avoidance of high-risk patients or procedures. Responses were recorded as none, rare, sometimes or always [10]. For analytical purposes, DM prevalence was defined as reporting at least one (≥ 1) defensive behavior among the listed practices.

Section 3 examined the causes of defensive medicine practices using structured questionnaire items. Participants were asked to indicate whether specific factors contributed to their practice of defensive medicine, including fear of compensation claims, fear of medical litigation, fear of disciplinary sanctions, and previous involvement in medical litigation. Multiple responses were allowed [10, 12]. The full English-language version of the questionnaire used in this study is provided as Supplementary File 1.

A pilot study was conducted among 34 physicians (10% of the total sample) to assess the clarity, validity, and reliability of the questionnaire and to ensure the appropriateness of the terminology used. No modifications were required following pilot testing, indicating adequate face validity and functional reliability of the instrument; therefore, pilot responses were retained in the final dataset.

Given that the questionnaire comprised multiple distinct constructs (e.g., defensive medicine behaviors and workplace violence exposure), internal consistency measures such as Cronbach’s alpha were not considered appropriate for the overall instrument. Instead, reliability was addressed through pilot testing to ensure clarity, stability, and interpretability of responses. This approach is consistent with recommendations for heterogeneous instruments assessing conceptually distinct domains.

### Statistical analysis

The collected data were coded, entered, and analyzed using EPI Info software version 6.02 and the Statistical Package for the Social Sciences (SPSS) version 25. Qualitative variables were summarized as frequencies and percentages, while quantitative variables were expressed as medians and interquartile ranges.

Comparisons between qualitative variables were performed using the Chi-square (χ²) test, with Fisher’s exact test applied when the expected cell counts were less than 5. A *p*-value < 0.05 was considered statistically significant. Binary logistic regression analysis was performed to identify factors independently associated with defensive medicine practices. Variables showing a statistically significant association in the bivariate analysis (*p* < 0.05) were entered into the multivariable logistic regression model. Multicollinearity among independent variables was assessed using the variance inflation factor (VIF) before model construction. Model goodness-of-fit was evaluated using the Hosmer–Lemeshow test, and adjusted odds ratios (AORs) with 95% confidence intervals (CIs) were reported. The EPV was 64.6 (323 events and 5 predictors), indicating an adequate number of events per predictor and supporting the stability of the regression estimates.

## Results

### Demographic and occupational characteristics of the studied physicians

A total of 340 physicians were included in the study. Nearly half of the participants were 31 years old or more (54.7%, interquartile range: 29–35 years). Males constituted more than half of the sample (56.8%). Regarding marital status, 68.5% of all participants were married. Physicians from medical specialties comprised 52.1% of participants, while those from surgical specialties accounted for 47.9%. Moreover, 53.2% worked for 6 years or more, and nearly half of the participants (50.9%) were working for 10 h or more per day. Detailed demographic and occupational characteristics are presented in Table 1.

**Table 1:** Demographic and occupational characteristics of the studied physicians (*N* = 340)

Characteristic	Value	*n* (%)
Age (years)		
Median	31	
Interquartile range	29–35	
< 31	—	154 (45.3)
≥ 31	—	186 (54.7)
Sex	—	
Male	—	193 (56.8)
Female	—	147 (43.2)
Marital status	—	
Married	—	233 (68.5)
Single	—	105 (30.9)
Divorced	—	2 (0.6)
Specialty	—	
Surgical	—	163 (47.9)
General surgery		45 (27.6)
Urology		33 (20.2)
Orthopedic surgery		18 (11.1)
ENT		16 (9.8)
Anesthesia		17 (10.4)
Obstetrics and gynecology		34 (20.9)
Medical	—	177 (52.1)
Internal medicine		49 (27.7)
Cardiology		22 (12.4)
Dermatology		14 (7.9)
Family medicine		30 (16.9)
Pediatrics		43 (24.4)
Psychiatry		19 (10.7)
Total length of time in the profession (years)		
Median	6	
Interquartile range	3–10	
< 6 years	—	159 (46.8)
≥ 6 years	—	181 (53.2)
Number of working hours/day		
Median	10	
Interquartile range	8–12	
< 10 h (median)	—	167 (49.1)
≥ 10 h (median)	—	173 (50.9)

### Prevalence of defensive medicine practices

**Fig. 1:**
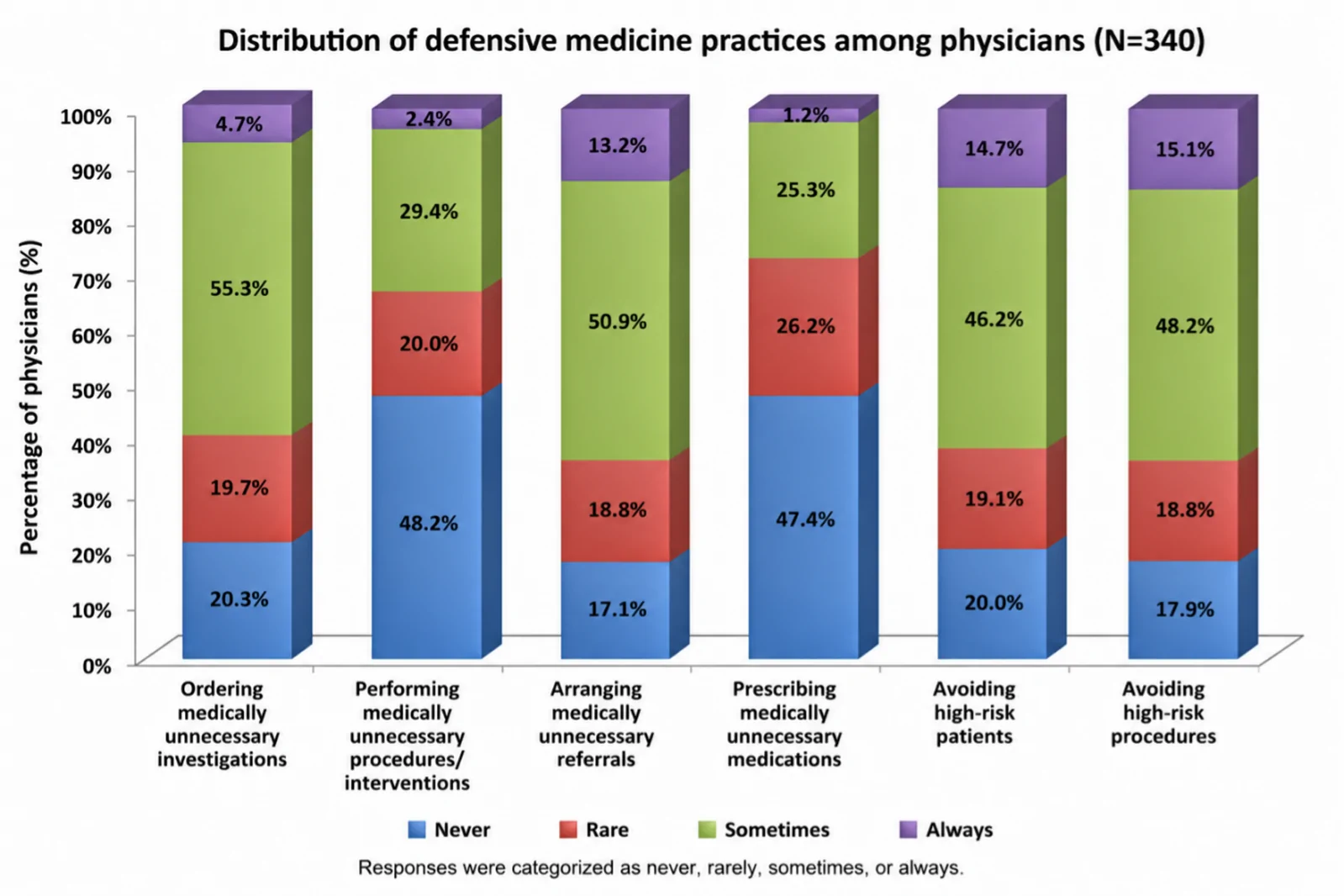
Distribution of DM practices among physicians (*N* = 340)

Overall, 95.0% (323/340) of the participating physicians reported engaging in at least one defensive medicine (DM) practice during their professional careers. Among positive defensive medicine behaviors, arranging unnecessary referrals was the most commonly reported practice (82.9%), followed by ordering unnecessary investigations (79.7%) and prescribing unnecessary medications (52.6%). Performing unnecessary procedures or interventions was the least frequently reported positive defensive behavior (51.8%). Regarding negative defensive medicine practices, 82.1% of physicians reported avoiding high-risk procedures, while 80.0% reported avoiding high-risk patients (Fig. 1).

The stacked bars represent the frequency of reported behaviors, including ordering unnecessary tests, carrying out unnecessary procedures, arranging unnecessary referrals, prescribing unnecessary medications, avoiding high-risk patients, and avoiding high-risk procedures. Responses are categorized as *Never*, *Rare*, *Sometimes*, and *Always*, and values are expressed as percentages.

### Reasons for practicing DM

According to the participants’ opinion, the most commonly reported reasons for practicing DM were fear of medical litigation (68.8%) and fear of disciplinary sanctions (49.7%). Prior personal experience with medical litigation was the least reported reason (25.6%). Multiple responses were allowed. The distribution of reported reasons is shown in Table 2.

**Table 2:** Reasons for DM practice among physicians (*N* = 340)

Reason	*n* (%)
Fear of medical litigation	234 (68.8)
Fear of disciplinary sanctions	169 (49.7)
A colleague’s prior history of facing medical litigation	148 (43.6)
Fear of negative publicity and loss of image	126 (37.1)
Fear of making a compensation request	118 (34.7)
Prior personal experience in medical litigation	87 (25.6)

Multiple responses were allowed; percentages do not sum to 100

### Factors associated with DM practice

Among the studied physicians, 76.8% (261/340) reported being physically attacked and/or verbally abused in the workplace in the past 12 months. On assessing factors associated with DM practice, avoiding DM practices was significantly more prevalent among physicians aged ≥ 31 years compared with younger physicians (*p* = 0.004), among males compared with females (*p* = 0.02), among those with ≥ 6 years of professional experience (*p* = 0.003), and among physicians working < 10 h per day (*p* = 0.001). Exposure to physical and/or verbal workplace violence was significantly associated with practicing DM (*p* < 0.001). No statistically significant association was observed with marital status, specialty, or prior involvement in medical litigation (Table 3).

**Table 3:** Practice of DM among the sampled physicians

Characteristic	No DM *n* = 17(%)	DM Practicing *n* = 323(%)	*P*-value
Age (years)			0.004*^^^^
< 31	2 (11.8)	152 (47.1)	
≥ 31	15 (88.2)	171 (52.9)	
Sex			0.02*^^^
Male	12 (70.6)	135 (41.8)	
Female	5 (29.4)	188 (58.2)	
Marital status			0.88^^^^
Married	11 (64.7)	222 (68.7)	
Single	6 (35.3)	99 (30.7)	
Divorced	0 (0)	2 (0.06)	
Specialty			0.12^^^
Surgical	5 (29.4)	158 (48.9)	
Medical	12 (70.6)	165 (51.1)	
Years in profession			0.003*^^^^
< 6 years	2 (11.8)	157 (48.6)	
≥ 6 years	15 (88.2)	166 (51.4)	
Working hours/day			0.001*^^^^
< 10	15 (88.2)	152 (47.1)	
≥ 10	2 (11.8)	171 (52.9)	
Exposure to workplace violence			< 0.001**^^^
Yes	7 (41.2)	254 (78.6)	
No	10 (58.8)	69 (21.4)	
Involvement in medical litigation			0.07^^^^
Yes	3 (17.6)	49 (15.2)	
No	14 (82.4)	274 (84.8)	

*P < 0.05 significant

**P < 0.001 highly significant

^*P*−value for Chi−square test

^^Fischer Exact test

### Predictors of DM practice

Binary logistic regression analysis identified exposure to physical and/or verbal workplace violence as an independent predictor of DM practice (adjusted odds ratio [AOR] = 3.28; 95% CI: 1.01–10.66; *p* = 0.048). Other variables, including age, sex, years of experience, and daily working hours, were not statistically significant predictors in the multivariable model. The model demonstrated adequate goodness-of-fit according to the Hosmer–Lemeshow test (χ² = 4.322, df = 6, *p* = 0.633) (Table 4).

**Table 4:** Logistic regression analysis for predictors of DM practices

Predictor	Β	(AOR)	95% CI	*P*-value
Age (< 31 years)	-0.875	0.417	0.053–3.277	0.406
Sex (Female)	-0.243	0.784	0.222–2.766	0.705
Years in profession (< 6 years)	-0.690	0.501	0.063–3.984	0.514
Working hours/day (≥ 10 h)	1.084	2.958	0.571–15.323	0.196
Exposure to workplace violence (Yes)	1.188	3.28	1.010–10.66	0.048*

Model fit assessed using Hosmer–Lemeshow test (χ² = 4.322, df = 6, *p* = 0.633)

*P< 0.05 significant

### DM practices by specialty

A statistically significant difference was observed between DM practices and physician specialty. Surgical physicians were significantly more likely to perform unnecessary procedures (*p* = 0.04), avoid high-risk patients (*p* = 0.03), and avoid high-risk procedures (*p* = 0.004). In contrast, physicians in medical specialties were more likely to order unnecessary investigations (*p* = 0.02). No significant differences were observed between specialties in terms of unnecessary referrals or prescribing unnecessary medications (Table 5).

**Table 5:** DM practices among surgical vs. medical physicians

DM practice	Surgical *n* = 163	Medical *n* = 177	*P*-value
Ordering unnecessary tests			0.02*^^^
Never	39 (23.9)	26 (14.7)	
Rarely	22 (13.5)	43 (24.2)	
Occasional	93 (57.1)	101 (57.1)	
Always	9 (5.5)	7 (4.0)	
Carrying out unnecessary procedures			0.04*^^^^
Never	66 (40.5)	98 (55.4)	
Rarely	40 (24.5)	28 (15.8)	
Occasional	52 (31.9)	48 (27.1)	
Always	5 (3.1)	3 (1.7)	
Arranging unnecessary referrals			0.29^*^*^
Never	23 (14.1)	35 (19.8)	
Rarely	27 (16.6)	37 (20.9)	
Occasional	90 (55.2)	83 (46.9)	
Always	23 (14.1)	22 (12.4)	
Prescribing unnecessary medications			0.80^*^^*^
Never	77 (47.2)	84 (47.5)	
Rarely	46 (28.2)	43 (24.3)	
Occasional	38 (23.4)	48 (27.1)	
Always	2 (1.2)	2 (1.1)	
Avoiding high-risk patients			0.03*^^^
Never	22 (13.5)	46 (26.0)	
Rarely	31 (19.0)	34 (19.2)	
Occasional	85 (52.2)	72 (40.7)	
Always	25 (15.3)	25 (14.1)	
Avoiding high-risk procedures			0.004*^^^
Never	23 (14.1)	38 (21.5)	
Rarely	27 (16.6)	37 (20.9)	
Occasional	95 (58.3)	69 (39.0)	
Always	18 (11.0)	33 (18.6)	

*P < 0.05 is significant

^*P*−value for Chi−square test

^^ Fischer Exact test

### Involvement in medical litigation by specialty

**Fig. 2:**
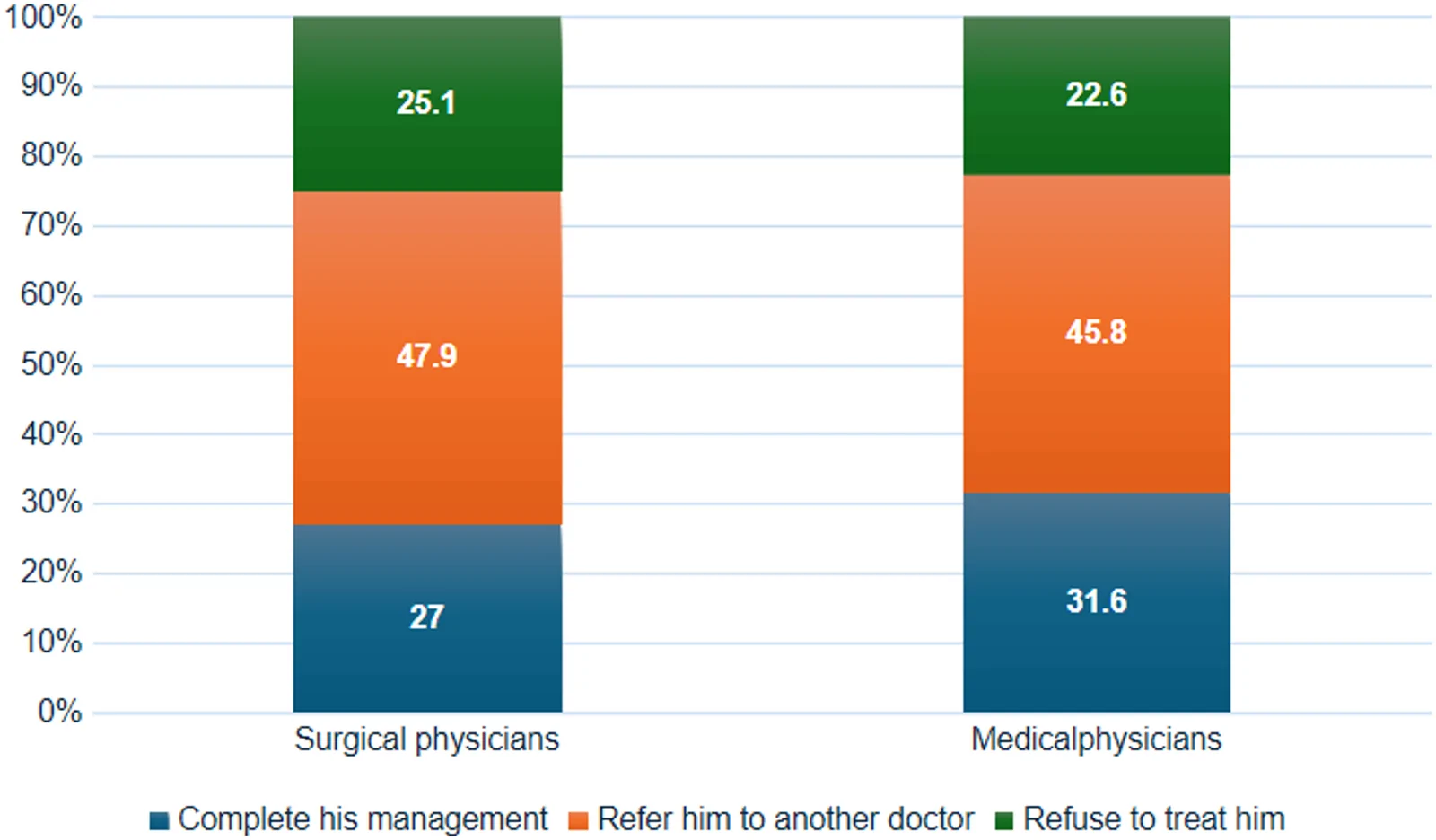
Distribution of surgical vs. medical physicians regarding their reactions to patient complaints (*N* = 163 vs. 177)

Surgical physicians reported significantly higher involvement of colleagues in medical litigation compared with medical physicians (*p* < 0.001) and a significantly greater tendency to consider working abroad due to fear of litigation (*p* = 0.006). Conversely, willingness to accept patients previously involved in medical litigation was significantly higher among physicians in medical specialties (*p* = 0.005) (Table 6). There was no significant difference between surgical vs. medical physicians regarding their reaction if a patient complained them (*p* = 0.63) where physicians’ reactions are clarified in Fig. 2.

**Table 6:** Medical vs. surgical physicians’ involvement in medical litigation and its professional impact

Experience with medical litigation	Medical *n* = 177(%)	Surgical *n* = 163(%)	*P*-value
Involvement in medical litigation	27 (15.3)	25 (15.3)	0.98
Involvement of colleagues in medical litigation	121 (68.4)	145 (89)	< 0.001**
Willing to accept patients previously involved in litigation	55 (31.1)	29 (17.8)	0.005*
Medical litigation increases the desire to work abroad	79 (44.6)	97 (59.5)	0.006

*P < 0.05 significant; **P < 0.001 highly significant

## Discussion

Defensive medicine is a well‑recognized and persistent global phenomenon, particularly in healthcare environments marked by high medico‑legal pressure, uncertainty, and limited institutional safeguards. The present study demonstrates a notably high prevalence of defensive medicine among physicians working in a tertiary academic healthcare setting in Zagazig, Egypt—a finding consistent with prior national and international research [10, 15, 16].

The prevalence observed in this study (95%), reflecting behaviors reported across physicians’ entire professional careers, lies at the upper boundary of global estimates, which range from approximately 60% to 98% [17, 18]. This finding should be interpreted in light of the study’s operational definition, which classified physicians as practicing defensive medicine if they reported at least one (≥ 1) defensive behavior at any point in their careers. This inclusive definition increases sensitivity and may partially overestimate prevalence compared with studies using stricter thresholds or frequency-based criteria.

In addition, the timeframe used to assess defensive medicine in this study differs from that adopted in many previous investigations, which often focus on a defined recent period (e.g., the preceding 12 months). By contrast, the present study employed a lifetime recall approach to capture cumulative exposure. While this broader temporal framework enhances the ability to detect any occurrence of defensive behavior, it may also introduce recall bias and further contribute to elevated prevalence estimates. Moreover, it limits direct comparability with studies using shorter recall periods. Therefore, the findings should be interpreted with caution when compared across studies employing different temporal definitions. Despite this limitation, the lifetime approach provides valuable insight into the overall tendency toward defensive practice rather than short-term behavior.

High rates worldwide suggest defensive medicine stems from universal pressures rather than local differences. These include fear of litigation, diagnostic uncertainty, increased patient expectations, and a global emphasis on blame avoidance over value‑based, patient‑centered care [17, 18]. Rather than reflecting true variability in clinical standards, this apparent consistency suggests that defensive medicine is driven by common underlying forces across contexts, including fear of litigation, diagnostic uncertainty, increasing patient expectations, and a global culture of accountability that prioritizes error avoidance over value-based care [18, 19]. Even in highly resourced systems with established clinical guidelines, these factors continue to sustain defensive practice patterns.

Notably, the prevalence of negative defensive practices—avoiding high‑risk patients or procedures (fig. 1)—was higher than that reported in several prior Egyptian and regional studies, where such avoidance, though frequent, was less extensive [10, 16]. Although defensive practices may appear protective from the physician’s perspective, they can generate significant clinical, financial, and ethical consequences. Ordering unnecessary investigations or referrals increases healthcare costs, prolongs patient pathways, and introduces avoidable risks such as radiation exposure and procedural complications [11, 19]. Conversely, avoiding high‑risk but clinically appropriate interventions may delay diagnosis or appropriate management, thereby compromising patient safety and outcomes. Importantly, such behaviors should not be interpreted as negligence or lack of professionalism; rather, they reflect physicians’ attempts to minimize medico‑legal risk within a system perceived as punitive, uncertain, and inadequately protective.

Fear of medical litigation was the most common driver of defensive medicine in this study, consistent with findings from Egypt, Italy, and multiple international contexts [10, 19, 20]. In settings where malpractice claims are increasingly visible and legal proceedings are perceived as adversarial, physicians may prioritize self‑protection over clinical intuition. Limited access to legal support and indemnity coverage further exacerbates this fear, particularly in tertiary hospitals that routinely manage complex, high‑risk cases [5].

Comparative international research highlights structural and socioeconomic variations in defensive medicine. Studies from low- and middle-income African countries report lower levels of defensive practice among neurosurgeons compared with high-income settings, likely due to economic constraints, cultural expectations, and weaker medico‑legal pressures [21]. However, a systematic review across 23 countries found an overall defensive medicine prevalence of 75.8%, with the highest rates in African and lower-middle-income regions (up to 89%) and among specialties such as anesthesiology [22].These differences highlight the interaction of legal infrastructure, resources, cultural norms, and specialty‑specific risks.

Gender differences were evident in the current study, with male physicians practicing defensive medicine less frequently than female physicians. In the Egyptian context, female physicians may perceive a higher personal or reputational cost for medical errors, leading to a greater reliance on “positive” defensive behaviors like ordering extra tests to ensure clinical thoroughness. Institutional and Cultural Scrutiny: Female doctors may face unique professional expectations or more intense scrutiny from both patients and peers, which can amplify the fear of litigation or disciplinary action—the primary drivers of DM in this study. Although evidence remains mixed, possible explanations include differences in risk perception, levels of scrutiny, and professional expectations experienced by women in clinical roles [23, 24].Further qualitative and longitudinal research is needed to clarify the mechanisms underlying gender‑based differences in defensive behavior.

Age and seniority were also important determinants: older and more experienced physicians were less likely to engage in defensive medicine, likely due to increased confidence, clinical competence, and experience‑based risk assessment [25] These findings This finding supports previous observations in Egypt by Arafa et al. (2023) that younger physicians are more susceptible to practicing defensive medicine [10].

Notably, although the model showed acceptable goodness-of-fit based on the Hosmer–Lemeshow test, only one predictor was statistically significant, with a wide confidence interval (AOR = 3.28; 95% CI: 1.01–10.66), indicating limited precision. Therefore, the findings should be interpreted cautiously, the association reached statistical significance, and aligns with existing evidence linking exposure to aggression with fear, emotional exhaustion, and risk‑averse clinical decision-making [11, 26]. In such contexts, defensive medicine may function as a coping mechanism to prevent conflict escalation rather than a purely medical judgment. Prior studies have similarly shown that workplace violence contributes to psychological distress, burnout, decreased job satisfaction, and poorer clinical judgment [26].

Reports indicate that workplace violence is significantly more prevalent in Arab countries in study on violence exposure of health care workers in Saudi public hospitals (67.4%) of respondents reported they were victims of violence in the previous 12 months, compared to Western settings such as the United States, the United Kingdom, and Japan [27–30]. Systematic reviews estimate that 20% to more than 60% of healthcare workers worldwide experience violence annually, with even higher rates in emergency and public hospitals [27, 31]. Contributing factors include overcrowded facilities, staff shortages, insufficient security enforcement, limited public awareness of healthcare workers’ rights, and cultural norms permitting verbal aggression toward professionals [29, 32]. Weak legal deterrents may exacerbate the problem.

Regarding specialty-based differences, surgical physicians in this study were more likely to perform unnecessary procedures and avoid high-risk patients and interventions, while medical physicians were more inclined to order unnecessary diagnostic tests. The observed difference between surgeons and physicians can be interpreted in light of decision-making under uncertainty and medicolegal risk. These findings align with evidence that surgical specialties carry a higher medico‑legal risk due to the immediate and visible nature of procedural outcomes, so they may adopt avoidance-type behaviors, such as refraining from high-risk cases, to minimize liability exposure. While non-surgical professionals may resort to over-testing or excessive documentation to avoid missed diagnosis as a means of self-protection against malpractice claims [3, 33–35]. Accordingly, surgeons may adopt more explicit defensive behaviors to avoid litigation.

Taken together, these findings underscore that defensive medicine is not merely an individual physician behavior but a systemic response to the interplay of medico‑legal pressure, workplace violence, cultural expectations, and institutional deficiencies. Efforts to reduce defensive practices require addressing these broader structural factors. Prior research emphasizes the need for legal reform, improved institutional protective policies, and enhanced communication strategies to reduce litigation‑related stress [18, 36].

Although the current study did not directly examine institutional policies, legal frameworks, or training programs, the high prevalence of defensive medicine and its association with fear of litigation and workplace violence highlight urgent areas for intervention [18, 36].

Hospitals can mitigate defensive medicine by enforcing zero-tolerance workplace violence policies and de-escalation training to reduce the fear-driven behaviors identified in the study. Systemically, the 2024 Medical Liability Law should decriminalize medical errors and establish specialized adjudication committees to alleviate the litigation anxiety reported by 68.8% of physicians. These reforms aim to shift Egypt’s healthcare from a “blame culture” toward a “just culture” where institutional protections replace self-protective clinical overutilization.

### Study strengths, limitations, and recommendations

A key strength of this study is its conduct within a tertiary referral center encompassing a wide range of medical and surgical specialties, providing a robust representation of defensive medicine practices in complex clinical environments. However, several limitations must be acknowledged.

First, the cross-sectional design precludes causal inference. Observed associations—particularly between workplace violence and defensive behavior—should be interpreted as correlational. Reverse causation and residual confounding from unmeasured variables cannot be excluded. Second, conducting the study in a single tertiary academic center may limit generalizability to other Egyptian healthcare settings. Although stratified sampling improved internal representativeness, differences in institutional culture, case mix, and medico‑legal climate across hospitals may influence defensive behaviors. Multicenter studies are needed for broader generalizability.

Third, reliance on a self-administered questionnaire introduces risks of recall bias, reporting bias, and social desirability bias. Additionally, different recall periods were used for key variables: defensive medicine behaviors were assessed across physicians’ entire careers, whereas workplace violence exposure was limited to the preceding 12 months. This discrepancy may affect comparability and should be considered when interpreting associations. Furthermore, the use of a broad operational definition (≥ 1 behavior) and self-reported measures may have contributed to the overestimation of defensive medicine prevalence.

Self-selection bias is also possible: physicians with personal experiences of workplace violence or heightened litigation concerns may have been more motivated to participate, potentially inflating prevalence estimates. Finally, institutional, organizational, and legal factors that likely influence physicians’ decision‑making were not assessed.

Another limitation is studying workplace violence as a crude prevalence without studying its types or severity. So, future studies with validated workplace violence scales are suggested.

## Conclusion

This study demonstrates a high prevalence of defensive medicine practices among physicians at a tertiary academic hospital in Egypt. Defensive behaviors were found to be associated with several individual and workplace-related factors, including fear of litigation, exposure to workplace violence, physician age, gender, and specialty. While fear of medical litigation and disciplinary sanctions were commonly reported by participants as perceived drivers of defensive practices, these factors were not directly analyzed as predictors and should therefore be interpreted as contextual influences rather than causal determinants. Overall, the findings underscore the need for interventions targeting workplace safety and supportive clinical environments to reduce defensive practices and improve the quality of patient care.

## Supplementary Information

Supplementary Material 1.

## Data Availability

The datasets generated and/or analyzed during the current study are not publicly available due to ethical and privacy considerations, but are available from the corresponding author upon reasonable request.
