# Supplementary material for: Defensive medicine practices and workplace violence among physicians in tertiary healthcare settings in Zagazig, Egypt: a cross-sectional study
Source: BMC Public Health. 2026 Sep 8;26:2697. doi: 10.1186/s12889-026-29209-9 (PMC13621763; doi:10.1186/s12889-026-29209-9)
Supplement: Supplementary file 1 [file 12889_2026_29209_MOESM1_ESM.docx]

**Defensive Medicine Practices and Workplace Violence among Physicians in Tertiary Healthcare Settings in Egypt: A Cross-Sectional Study**

This survey aims to determine the prevalence of both positive and negative DM practices among physicians and to identify the underlying causes of these practices. All collected data will be used for research purposes only, and no identifying information is requested. Participation is fully voluntary, and continuing with the survey indicates your consent. Please answer according to what best reflects your situation.

**Section 1**

**Sociodemographic and occupational data**

1. **Age (in years)** ……………….

2. **Sex**

Female

Male

3. **Marital status**

Married

Unmarried

Divorced

Widow

4. **Specialty**

Medical

Surgical

5. **Years of experience**

*………………

6. **Working hours/day**

*……………….

7. **Were you exposed to physical and/or verbal workplace violence?**

Yes

No

**Section 2**

**Defensive medicine practice**

8. **Do you order tests that are probably not clinically indicated to avoid possible legal consequences?**

Never

Rarely

Sometimes

Always

9. **Do you carry out interventions or procedures that are probably unnecessary to avoid possible legal consequences?**

Never

Rarely

Sometimes

Always

10. **Do you arrange unnecessary referrals to other specialties to avoid possible legal consequences?**

Never

Rarely

Sometimes

Always

11. **Do you prescribe unnecessary medications to avoid possible legal consequences?**

Never

Rarely

Sometimes

Always

12. **Do you avoid high-risk patients to avoid possible legal consequences in the case of complications?**

Never

Rarely

Sometimes

Always

13. **Do you avoid high-risk procedures to avoid possible legal consequences in the case of complications?**

Never

Rarely

Sometimes

Always

**Section 3**

**The reasons for defensive behaviors and involvement in medical litigation**

14. **What factors influenced the behaviors indicated above? (more than one answer available)**

Fear of a request for compensation

Fear of medical legal litigation

Fear of disciplinary sanctions

Fear of negative publicity, loss of image

Previous personal experience of medical-legal litigation

Previous experience of a colleague being subject to medical-legal litigation

15. **Have you been involved in medical litigation?**

Yes

No

16. **Has any of your colleagues ever been involved in medical litigation?**

Yes

No

17. **Are you willing to accept patients who were previously involved in medical litigation?**

Yes

No

18. **If a patient complained against you, what is your reaction?**

Complete his management.

Refer him to another doctor.

Refuse to treat him

19**. Does your fear of medical litigation increase your desire to work abroad?**

Yes

No
